# A life marked by early school leaving: gendered working life paths linked to health and well-being over 40 years

**DOI:** 10.1186/s12889-024-19447-0

**Published:** 2024-07-23

**Authors:** Anneli Silvén Hagström, Anne Hammarström

**Affiliations:** 1 Department of Social Work, Stockholm University, Stockholm, Sweden; 2 Institute of Environmental Medicine, Karolinska Institutet, Stockholm, Sweden; 3 Department of Epidemiology and Global Health, Umeå University, Umeå, Sweden

**Keywords:** Gender, Health, Labour market, Longitudinal study, Narrative, School Leaver, NEET, Unemployment, Youth

## Abstract

**Background:**

There is increasing awareness of the need to analyse symptoms of mental ill-health among early school leavers. Dropping out of compulsory education limits access to the labour market and education and could be related to deteriorating mental health over the course of a lifetime. The aim of this longitudinal study is to explore how early school leavers not in education, employment or training (NEET) narrate their working life trajectories linked to health, agency and gender relations.

**Methods:**

Twelve early school leavers in the Swedish Northern Cohort (six women and six men) were interviewed over 40 years about their working life and health. Their life stories were analysed using structural narrative analysis to examine the evolution of their working life paths and to identify commonalities, variations and gendered patterns.

**Results:**

All the participants started in the same position of “an unhealthy gendered working life in youth due to NEET status”. Subsequently, three distinct working life paths evolved: “a precarious gendered working life with negative health implications”, “a stable gendered working life in health challenging jobs” and “a self-realising gendered working life with improved health”. Agency was negotiated through struggle narratives, survival narratives, coping narratives and redemption narratives.

**Conclusions:**

Even in a welfare regime like Sweden’s in the early 1980s, early school leavers not in education, employment or training experienced class-related and gendered working and living conditions, which created unequal conditions for health. Despite Sweden’s active labour market policies and their own practices of agency, the participants still ended up NEET and with precarious working life paths. Labour market policies should prioritise reducing unemployment, combating precarious employment, creating job opportunities, providing training and subsidised employment in healthy environments, and offering grants to re-enter further education. Our study highlights the need for further analyses of the contextual and gendered expressions of health among early school leavers throughout their lifetime, and of individual agency in various contexts for overcoming adversities.

## Background

Early school leaving is linked to increased risks of long-term unemployment, social exclusion and poverty [1–3]. Limited educational attainment narrows job prospects, increasing the risk of getting precarious, physically demanding, and low-paid jobs, especially for young people [4–6]. There is a growing awareness of the need to analyse how symptoms of mental health are related to leaving school early. The only meta-analyses in the field [7] concerning the mental health of young people not in education, employment, or training (NEETs) examined only 24 quantitative papers. While these identified relations between mental ill-health, problems of substance misuse and being NEET, they could not ascertain the direction of causality of these relationships [5]. Early youth was found to be a critical period marked by heightened prevalence of anxiety problems, alcohol consumption, and psychological distress. Being NEET during this period was shown to increase vulnerability to NEET status later on. However, significant knowledge gaps were identified, highlighting the necessity for both contextual and gendered analyses to accurately assess health outcomes [7]. Both gaps are preferably analysed using qualitative methods that investigate the subjective experiences of individuals who leave school early and find themselves in a NEET position. Longitudinal studies are crucial for understanding how ill-health associated with early school leaving develops over time and identifying ways to mitigate it. Such studies among young people in NEET are particularly important, as they provide repeated opportunities to discuss their working life trajectories and symptoms of mental ill-health. They offer insights into how young people interpret their working lives in relation to mental health and evaluate their scope for action during life. Such knowledge is crucial for informing the development of tailored social policies aiming at preventing poor mental health among young people related to early school leaving and addressing the needs of young people with NEET status.

This study adopts both a structural perspective and an individual agency-focused approach, as suggested by Ruth Lister [8], to investigate young people’s narrated working life trajectories, which include experiences of marginalisation and poverty. This dual approach recognises that disadvantaged conditions stem from societal resource distribution and public social policy, but also explores how individuals navigate their situations using their own agency. The study also applies Raewyn Connell’s relational theory [9, 10] to examine how the societal gender order, constructed by multiple ideas of femininities and masculinities in various historical and societal contexts, creates gendered conditions in the labour market and romantic relationships. Gender relations in working life construct a gender-segregated labour market, which divides men and women into different sectors and hierarchies, thereby establishing gendered working conditions. In private life, gender relations are constructed around power dynamics; even in more gender equal countries like Sweden, women still primarily bear responsibility for the home and family [11]. Femininities and masculinities are constructed in the context of the societal gender order, thereby creating and maintaining male domination. The aim of this longitudinal study was to explore how early school leavers with NEET status narrated their working life trajectories linked to health, gender relations, and agency.

## Methods

### Setting

The interviews for this qualitative study were performed with a group of early school leavers in the Northern Sweden Cohort [12]. The cohort was created in 1981 in a medium-sized industrial town of about 70,000 inhabitants in northern Sweden. It comprised all the pupils (*n* = 1083) in their final year of compulsory schooling in the municipality. The cohort has been shown to be representative of Sweden regarding self-rated health conditions, sociodemographic factors and labour market conditions, apart from the very high unemployment rate in the 1980s [12]. When the cohort was created, the unemployment rate in the region was twice the average for the rest of Sweden. In addition, workforce migration from Finland was somewhat larger than in the rest of the country. The labour market was dominated by manufacturing and mineral extraction, a steel company and a large harbour. Other major employers were the public sector and a technical university. The social democrats had dominated local government for several decades.

To tackle the growing rate of youth unemployment, the Swedish government introduced active labour market policy measures for young people at the beginning of the 1980s. These measures included educational and vocational activities directed at unemployed young people with the objective that no one aged 18 years or under should be outside of education or employment. Subsidised employment, involving work tasks that did not compete with regular jobs, was the most common labour market measure for young people in the early 1980s [3]. Labour market policy measures for people under the age of 18 were financed by the state, providing study assistance instead of a salary. These were intended to guarantee activity for 8 h a day for 40 weeks for unemployed persons in the age group 16–18 years. In 1982, the period of reimbursement was increased beyond 40 weeks. In 1984, a law was introduced stating that unemployed 18- to 19-year- olds should participate in labour market activities for at least four hours a day in return for a minimum wage ( [13], p. 4). This meant that, by international standards, Sweden had a highly active labour market policy aimed at young people in the early 1980s. For the participants in our study, these active labour market policy programmes meant that they did not become long-term unemployed in their youth, but instead moved between unemployment, various labour market programmes and short periods of temporary employment. However, lack of resources linked to regional differences in the number of unemployed could be a reason why the cohort participants became unemployed.

### Participants

The participants in this study were selected from the Northern Swedish Cohort, which includes all pupils in their last year of compulsory schooling in a medium-sized town in northern Sweden in 1981. All the participants (nine girls and eleven boys) who had dropped out of compulsory school or decided to leave school after compulsory schooling (ninth grade), and who were NEET one year later agreed to be included in this longitudinal interview study. One of the girls had left school due to pregnancy and was home with her child. After five years, two decided not to continue their participation (one girl and one boy). In addition, one girl did not want to be tape-recorded so the interviews could not be part of the narrative analysis. One girl and four boys have been part of the study throughout but as they moved around Sweden, there were too few tape-recorded interviews to be included in this paper. In total, 12 participants (6 women and 6 men) were included in the analysis for this study. Of these, nine had dropped out of compulsory schooling while three had completed it.

### Data collection

Personal interviews were carried out with all the participants over 40 years by the same PI (AH). The number of interviews varied in the individual cases between three and seven, with an average of 4–5 interviews; the first interview took place in 1982 or 1983, when the participants were 17–18 years old, and the follow-up interviews were conducted at various intervals until the participants reached middle age (around age 56). The PI is a GP and offered all the participants consultations about health problems if needed, which mainly led to recommendations to seek health care. Participants were typically interviewed briefly twice in the first 1–2 years, transitioning to more comprehensive interviews once every 10 years thereafter. The interviews were mainly conducted in the participants’ homes or in neutral settings such as libraries. In one case, a zoom interview was conducted due to the Covid-19 pandemic. The interviews followed a narrative structure [14], featuring open-ended questions that prompted participants to discuss their social, work-related, and health situations in detail. Follow-up questions were used to encourage narration, allowing for the collection of rich narrative material from all the participants except for one, who was less vocal. In the interviews, participants consistently re-evaluated the meaning of their lives and work situations in relation to their health and well-being. While the interviews primarily focused on the present, participants framed their narratives about current circumstances by reflecting on past life experiences and considering future aspirations. In the final interviews, participants were asked to narrate how their health had developed from early life, noting significant events to enhance life storytelling connected to health. The length of the interviews varied with age and ability to narrate and reflect in the context of a research interview. In particular, the initial interviews with some of the boys were quite short (around half an hour). The other interviews lasted initially for around one hour. The last two interviews were longer, lasting up to two or three hours. The interviews were recorded and transcribed verbatim. The total transcribed material comprised 1318 pages.

The narrative text produced in this study is regarded as a joint construction between the interviewer/researcher and the participants in a speech act consisting of questions and answers [15]. This is a particular discourse, as the storyteller adjusts narration to who s/he believes the interviewer to be and depending on how s/he wishes to present her-/himself in the context of the research. The narratives that come to light also depend on how the interviewer leads the interview. In this study, the trusting relationship that developed between the interviewer and the participants over time played a decisive role. Participants appreciated being followed by a supportive researcher, which influenced their willingness to share sensitive experiences.

### Ethical considerations

The primary ethical principle guiding this research has been to minimise the risks and burdens associated with participation. The longitudinal design raises ethical considerations regarding consent, participant care, confidentiality, and the representation of lives [16]. These considerations were continually addressed throughout the research process following ethical guidelines on informed consent. Maintaining contact with young people in vulnerable life situations for follow-up interviews poses additional ethical challenges, such as regulating the interviewer-informant relationship to avoid misguided expectations and overdependence [17]. Ensuring continuity in this relationship has been considered crucial to creating a safe space for discussion of sensitive topics and for the interviewer to enable support, although it has required the balancing of engagement with avoiding undue responsibility to empower participants. The interviewer’s involvement has been demonstrated through shorter follow-up telephone contacts between interviews and assistance when needed to contact the introductory programme, aimed at helping youth to get out of NEET.

### Narrative methodology

This study adopts a social constructionist and narrative perspective on the life course, recognising that humans naturally seek to make sense of their lives through the stories they construct about themselves [18]. Utilising McAdam’s [19] theories of narrative identity, men and women are viewed as autobiographical authors as they move through life, using life narrative as a tool for bringing order to their experiences. Personal narrative construction begins in childhood and evolves through youth and beyond, involving the translation of experiences into coherent stories. Narrativity, or the ability to narrate, develops through the life course and while youth is a time when narrative identities are primarily lived out in the present, narratives in mid-life tend to become more reflective and introspective [20]. This shift occurs as narrators become more aware of the finitude of life and concerns the psychological issue of wanting to create generativity and leave a meaningful legacy for future generations.

Life narratives typically feature a motivated protagonist (an agent) with a foreseen goal facing challenges that must be actively navigated, as the plot unfolds a temporal landscape encompassing the past, present and future [19]. Hence, when individuals are prompted to tell their life stories, in this case focused on their working life trajectories, it triggers their reflective thinking. This process involves constructing meaning from lived experience, evaluating current circumstances and considering opportunities to manage or change their situation in the future. Importantly, these narratives are shaped not only by individual cognition and social context, but also by broader structural power dynamics, gender relations, cultural norms and shared meanings.

### Data analysis

Structural narrative analysis is concerned with the exploration of narrative genres, which examine how stories are constructed and the meanings they convey [21]. In this study, longitudinal data underwent narrative analysis to reveal both the content and the structure of participants’ life stories. This approach focuses on both *what* is told and *how* it is told. The life stories were then configured into three distinct life paths based on their development over time, enhancing understanding of lived experiences [21]. The analysis was carried out in four steps:

1. *Initial reading and interpretation*: The interviews underwent multiple readings to explore participants’ perceptions of their evolving working life, social circumstances, health and well-being. Narratives were interpreted both as shorter sequences or plots and as cohesive life stories [21].
2. *Narrative thematic analysis*: Closest-to-data subthemes were identified and categorised into main themes. For example, subthemes such as “a boring daily life”, “lack of money” and “persistent stomach pain” were categorised into the following themes of “unemployment struggles”, “financial constraints” and “health episodes”.
3. *Identification of commonalities*,* variations and gendered patterns*: Commonalities, variations and gendered patterns were identified across cases, resulting in the structuring of three distinct working life paths.
4. *Focus on agency*: Participants’ agency for improved health was further analysed. In this context, agency is investigated as personal agency to manage or change one’s situation within structural constraints ( [8], pp.128–129). Participants negotiated agency through four narrative practices.

We use excerpts from participants’ life stories below to illustrate their configured life paths and narrative practices, and the implications for their health and well-being.

## Results and reflections

### Meaning construction of the decision to leave school

The nine participants who dropped out of compulsory schooling reflected on their dropping out differently over time as they constructed meaning from their experiences. When younger and recently out of school, some simply stated that they were “bored” or that school was just not a good fit. Others described challenges in school, including learning difficulties, peer bullying, lack of teacher support and motivation, or a desire for independence. In addition, many participants cited stressful family situations, such as parental mental health issues, alcohol misuse, violence, fractured family ties and loss of family members. It is plausible that such family challenges might have affected their ability to focus on schoolwork. Only in later interviews did some participants begin to see the links between family adversity, school challenges and their decision to drop out. They questioned why their basic needs were not recognised in school and why teachers were not more supportive, especially considering their teachers’ awareness of their family situations. However, most still blamed themselves for lacking the motivation to complete school, viewing their dropout as a personal failure.

**Table 1:** Narrated working life paths and narrative practices of agency

The point of departure for all participants	Three narrated working life paths	Narrative practices of agency
An unhealthy gendered working life in youth due to NEET status		*Struggle narratives* of managing unemployment, obtaining and enduring precarious jobs with psychosomatic outcomes
	I. A precarious gendered working life with negative health implications	*Survival narratives* of managing unemployment, obtaining and enduring precarious and health impairing jobs, as well as coping with social problems and chronic ill-health
	II. A stable gendered working life in health-challenging jobs	*Coping narratives* of enduring demanding work and chronic ill-health
	III. A self-realising gendered working life with improved health	*Redemption narratives* of achievements in overcoming adversity and forging successful and health-promoting careers

### The point of departure for all the participants: an unhealthy gendered working life in youth due to NEET status

A shared starting point and three distinct working life paths emerged from the life stories. Initially, all the participants expressed relief at having left school, but they soon faced precarious and unhealthy conditions marked by prolonged or recurring unemployment. Their working lives then developed along three gendered paths (see Table 1). Early on, all the life stories depicted a financially strained, monotonous life, which the participants related to somatic symptoms and mood changes. Participants highlighted their struggles to secure and manage jobs in physically and emotionally demanding, sometimes risky, gendered environments. They normalised these situations as typical for their age, viewing precarious jobs as stepping stones to future opportunities. However, they critically reflected on their vulnerability and their excessive individual responsibility to improve their circumstances without sufficient external support.

#### The struggle to cope with unemployment in youth

Before turning 18, participants experienced regular periods of unemployment while actively seeking jobs. Their narratives fluctuated between hope for employment and feelings of powerlessness and resignation. Most were financially dependent on their parents, many of whom, as previous research shows, also struggled due to low levels of education and low-paid jobs [22]. Unemployment affected their social lives, limiting possible activities and increasing their loneliness. They described loss of structure and meaning in everyday life. This monotony fuelled the participants’ desire for independence and a personal income. For instance, Sanna began skipping school in eighth grade due to low motivation and lack of teacher support. She eventually dropped out and became pregnant at a young age. By 18, prolonged unemployment was taking a toll on her mental health, exacerbated by the recent loss of her father. Despite attending short traineeships, she struggled to envisage a future.

You sit there and stare at the wall and try to find something [to do] to make time pass, until it’s night and time to go to bed. You don’t get anything done either. I don’t do much when I’m unemployed. I don’t care about anything. I just smoke one cigarette after another. You think: “Shit, if this is it, it might as well be over”.

Similarly, Jenny dropped out of school because she felt tired of it. At 17, she described feeling bored during periods of unemployment, spending her time sleeping, walking her dogs, reading and watching TV. Carola left school because she didn’t feel it was for her and preferred practical work. During unemployment, she cared for her siblings’ children and participated in short traineeships. Lotta left school in ninth grade and later attended a household management course but left because she wasn’t interested in it. She eventually moved out at 16 to live with her boyfriend in a remote cottage, spending her days alone while he worked.

It was terrible and I felt really bad. You tried to sleep as long as possible in the mornings, so that the day would pass faster. You couldn’t go anywhere and there was nothing to do. I used to call my mum to talk, but she got tired of me.

Lotta expressed feeling “like an idiot” and “useless” due to her unemployment. Siri, who experienced reading and writing difficulties, severe bullying and physical violence in school, wanted to leave “as soon as possible”. At 16, she complained about recurring stomach pain due to worries about the future.

It’s probably because I’m nervous and restless… I get nervous about the future and think: “Should I sit like this… won’t I get a job? Will I have to wait years and days until I get a job?” Sometimes I don’t think about it at all… I just think about having fun… going out with friends and enjoying life like that… When I think about it, I get depressed and think: “Am I going to be like the A-team [alcoholics] in town? What will I become?” You don’t know what to do… it’s so difficult because you can’t decide what to do in life.

Siri’s reflection highlights the common challenge of figuring out one’s path in youth and finding motivation. Like many other girls in her situation, she experienced various bodily symptoms such as distress, headaches, joint pain, heart palpitations and sleep issues. Limited in options, Siri, like others, sought jobs through the Introductory Programme and spent time with friends, often involving alcohol. Petra left school due to conflicts with teachers, feeling discriminated against and harassed. Unemployment weighed heavily on her, and she observed the aimlessness shared by many unemployed youths: “Teenagers sit in the park and chug beer. They’ve got nothing to do and, in the end, it doesn’t matter what you do”.

The unemployed young women in the study described how they used to take care of dogs and children for family and friends, sometimes in the company of an unemployed friend; several of them became mothers at a young age with older partners. On the other hand, the young men often spent time snowmobiling, fishing or working on cars with unemployed friends, and entered parenthood later in life. Thus, gendered romantic relations and related gendered living conditions were shaped at an early age, with women acting as the main caregivers [23]. Loneliness was a common theme in all the unemployment narratives, affecting participants’ outlook on life and was related to feelings of loss of meaning and low self-esteem.

#### The struggle to obtain employment

In line with unemployment benefit regulations, participants regularly visited their local employment agency from the age of 18 to apply for jobs, but faced strong competition from older, more experienced job seekers. Some managed to secure short job placements through local programmes, but these opportunities were limited and all the participants occasionally found themselves without work training. Given the high unemployment rate in the region, the agency commonly advised moving to larger cities in the south for better job prospects. This advice was emotionally challenging for the young teenagers. After leaving school due to unfair accusations of drug use, David briefly studied elsewhere but returned home, struggling with financial dependency and health issues. At 18, he engaged with the employment agency:

I’ve been to the [employment agency] and said that “Now you’ve got to find me a job!” But it doesn’t work, they just say “move!” all the time. “Nah” I say. I’ve been down looking for work in [a southern city] and [another southern city]. And it was probably nice, but moving there is another story. It’s so hard to start in a new place when you don’t know anything or anyone. Yes, it’s clear you’d miss home too… and my girlfriend isn’t too keen on moving like that.

David decided to stay in his hometown. He secured short traineeships through the youth opportunities programme but then became unemployed again, which led to worsening health problems and a “stressed heart”. He eventually considered returning to education, but his contact at the employment agency dissuaded him from doing so.

Sometimes I sit and think about friends that you see, or know a little bit, that they are almost 35 years old and have the same education as me and they are still unemployed. If it’s like that… hell no, I don’t want to be still unemployed when I’m 35… I told her at the [employment agency] that I was thinking about going to [municipal adult education] and everything, but no… she thinks it’s better that I move than start educating myself.

Siri recalled how she was given the same advice, to which her mother protested “Why should she move; she is only 16?! And where will she live?”. Fredrik could not leave home to seek work in other regions as he was the youngest child, responsible for caring for his aging and disabled parents. This is consistent with many other young carers’ life journeys, characterised by being tied to the childhood home environment in youth [24].

#### The struggle to manage precarious and gendered working conditions

Like others in precarious jobs, everyone valued having a job more than being unemployed, despite often describing poor working conditions [25]. Jimmy left school because he did not like being told what to do by the teachers. At 17, he expressed excitement about his new job as a lumberjack, relishing the opportunity to spend hours in the forest chopping down trees, even in challenging conditions with snow up his waist.

You get up around half past seven and then you work until… it depends on how long it takes, around nine, ten. That’ll be about 13 h a day. Well, it doesn’t have to be fun, I do it for work.

The young men entered physically strenuous jobs, in accordance with traditional masculine ideals. The jobs were informal, low-paid seasonal tasks for acquaintances obtained through personal connections, such as sawing wood and operating a tractor. Rickard was unable to pursue forestry studies as he had no driving licence due to a neurological disease. He also spent long hours chopping trees in the woods, working double shifts to save money for later periods of unemployment.

Both men and women in the study worked in physically demanding, low paid jobs. The young women more often began work in traditionally women-dominated jobs such as caregiving, cleaning or retail. This aligns with societal gendered norms, which expected women to take on caregiving and household responsibilities as part of a traditional, normative femininity [26]. This femininity contrasts with the ideal of hardworking masculinity, as seen in some of the men’s narratives where they adhere to the stereotype of working class young men who take any available job regardless of the hazards the tasks involve [23]. The young women shared their experiences of facing both gender and age discrimination. Sanna recounted instances where she was mistreated by employers and older colleagues. For instance, at a day care centre, she had to wear her own clothes while others had work attire, experienced unexplained reductions in her working hours and was assigned tasks others avoided. She also recalled feeling exploited at her job in a pet shop:

I liked it at first, but they were such bad colleagues… they were crazy. They didn’t think I did enough, although I struggled the whole day. They demanded too much of the young employees… all the things that we were expected to do. Unpacking goods for hours wasn’t fun. The youth employment centre wanted us to be trained to work the cash register, but no “we can’t let *any of them* stand at the checkout”. I had to clean out everything, and it wasn’t an ordinary bird cage but a room where they had bird breeding, so it was really dirty. The woman who had a permanent position complained about her back, so I had to drag all the shit upstairs, clean and vacuum like another dicky bird.

After a long period of unemployment Lotta eventually began to train as a welder, but she felt she was treated in a sexist way by the male employees: “Dirty old men sat there staring at you when you were welding. I burned myself all the time with welding fleas and got angry, and the old men kept looking, sniffing and were disgusting. So I quit”. Lotta recalled another incident during her time at a filling station when she fell ill with severe neck pain. Despite not requiring a medical certificate, her employer refused to give her sick leave. While both women and men faced tough working conditions, all the participants found that work renewed their sense of purpose and drive to navigate life, as precarious jobs were seen as potential stepping stones to advancement and more stable working conditions. Work contrasted with their negative experiences in school and of unemployment, which had negatively affected their self-esteem. However, precarious jobs have been likened to a trap, where the desire for advancement can ironically perpetuate poor working conditions and hinder progress towards more secure, fulfilling positions [27, 28].

During youth, the participants’ life stories were marked by diverse struggles: grappling with unemployment, searching for work and contending with precarious and unpleasant jobs. While they occasionally perceived themselves as victims of circumstances beyond their control, particularly during periods of unemployment that affected their health and well-being, their narratives also reflected active efforts to manage and improve their situations [29].

### I. A precarious gendered working life with negative health implications

Four participants, Jimmy, Rickard, Lotta and Sanna, shared narratives reflecting their lifelong struggle with insecure employment. Over time, they experienced social problems, illness and deteriorating health. The gendered labour market challenges described above continued. In addition, they faced challenges with romantic relations as well as with physical limitations and health problems. These diverse struggles contributed to an overarching survival narrative, where they exercised agency to manage their vulnerability and stress. Jimmy and Rickard continued to work in masculine coded jobs with a high risk of accidents and a lack of insurance. In his 30s, Jimmy described how he had repeatedly injured himself in workplace accidents.

First, I was hit by a truck at the steel company. I passed out and spent three days in hospital. Then I hurt myself in my janitorial job at the sports club, hit my back and leg, and fractured my tailbone. I fell from the ceiling; I was setting up goal cages because we were going to put them away until spring. I was in a hurry and slipped. Luckily, I fell between long nails sticking out of the floor, otherwise it could have been a lot worse.

In his 40s, Jimmy summarised his working life so far as having been meaningful, because he had always enjoyed working hard and for his body to feel that he had done “a proper day’s work”. However, his work-related health problems had worsened, including multiple accidents with repeated head traumas, which he believed negatively influenced his memory and gave him chronic pain. He had now been approved for a disability pension.

There’ve been plenty of accidents and in these accidents I’ve always, *always* hit my head and become unconscious. After that, I have a bad memory. It’s the short-term memory and sometimes… it’s like a block of time. There’s a little worry about how this is going to go, with the body, the legs, the aches, there’s a worry about that… I don’t want to be a cripple who can’t do what I want.

Jimmy’s previous meaning of fulfilling a “proper day’s work”, which shaped his self-perception as a physically strong man [23, 25], was challenged by his bodily limitations. Despite this, he coped by prioritising activities that brought him joy, like spending time with loved ones. His reflections underscored the significance of mental resilience and a positive outlook in confronting physical hurdles, stressing the importance of maintaining optimism. In his 50s, he looked back on his diverse work experiences with pride, noting his ongoing commitment to seeking employment despite obstacles. He disclosed battles with depression and alcohol misuse linked to past relationship and family challenges, which were described as having negatively affected his mental health. Finding a new love and the possibility of becoming a father again became a turning point, inspiring him to remain sober and prioritise self-care. His experiences also drove him to find purpose in supporting vulnerable teenagers in his community, turning his past hardships into a resource for aiding others. This resonates with McAdam’s theories [20] on midlife narratives, highlighting a generative aspect characterised by a dedication to future generations.

Rickard continued working in temporary jobs, forest clearing, wood cutting and truck driving, interspersed with periods of unemployment. He ended up living with his father in his childhood home and did not have a family of his own. He was tight-lipped about his life and health, but in his last interview, aged 56, he was still unemployed and suffering from various metabolic disorders in addition to the neurological disease he had had from a young age.

As a mother in her 30s, Lotta regretted her decision to leave school and blamed herself: “You were so stupid in school, so you screwed it all up. You should’ve straightened yourself out to get better grades, if only you’d had a stronger will”. In her final interview at age 56, Lotta was collecting disability pension due to mental ill-health, which stemmed from a history of repeated trauma. She had endured feeling neglected by her father, left home in her teens and escaped an abusive relationship, although she had faced a custody battle that separated her from one of her children. Following the passing of both parents, she grappled with exhaustion and depression, and received a neuropsychiatric diagnosis, alongside metabolic diseases. These health challenges permanently impaired her ability to work. Reflecting on her life, from the vantage point of early retirement, Lotta expressed pride in her resilience, crediting her inner strength: “I think I must have a psyche of steel because nothing has broken me yet. I’ve fallen down the stairs several times, but I’ve just climbed back up”. While finding relief in reconnecting with her estranged child and reuniting with her father, these events did not signify turning points as she continued to face new stressors impacting her health. She now expressed concern for another child and her struggles with abusive relationships.

In her 20s, Sanna attempted to resume her education but found it too challenging to balance studies with being a single mother. In her 30s, she found employment as a property manager, feeling valued and appreciated in this role despite her lack of formal education. However, a chronic medical diagnose restricted her ability to work effectively. She had faced stress and miscommunication with the employment agency while seeking support for a property maintenance course she wanted to take. Taking matters into her own hands, she independently reached out to the college, making a memorable impression as their first female applicant. Despite obstacles linked to medical disorders, financial strain and single parenthood, including supporting a dyslexic child in school, Sanna credited her ability to navigate these challenges to her determination: “I think it’s thanks to my stubbornness and persistence that I have survived. Sometimes everything has been dark as night, then you get so negative; but then I’ve learned that you put it aside and just let it be for a while, and then you take new springy steps”. At 56, Sanna had been on half-time disability pension for several years due to her medical diagnoses. Despite undergoing surgery, she still faced significant limitations in her daily life, prompting her to reassess her priorities and find value in ordinary moments, particularly with her grown children. As a single mother, she had juggled various responsibilities, including temporary jobs, while managing her poor health. Retiring was depicted a turning-point for improved well-being.

I want to *live*, and living is to *feel well*. To sit at home and have a cosy time with my children; we cook together and go to town and buy clothes and such… things that people think are ordinary are luxuries for us because we could never do that when they were small. Then it was everything from A to Z, work… and there was nothing else; we could never go anywhere because I was always sick.

Again, the men detailed a pattern of physically demanding and risky jobs, reflecting the ideal of a hardworking masculinity [23, 25]. The women also held physically demanding jobs but did not express the same pride in them as the men did. Normative femininities, constructed in relation to gendered caregiving [26], strained their health and well-being. Having the main responsibility for the family took precedence over their unstable careers. Chronic disorders were exacerbated by their gendered family and working lives, which further hampered their ability to establish stability in the job market.

Participants narrating this working life path witnessed how their lives were profoundly affected by their marginalised status in the labour market, a situation perpetuated over time. Initially hopeful of securing permanent employment and improved working conditions, their lived experiences told a different story. Echoing previous research, they found that their entry into precarious jobs only reinforced their marginalisation, failing to secure labour market attachment [27, 28]. Consistent with prior research, precarious employment perpetuates a cycle of ongoing insecurity, poor working conditions and deteriorating health status [30]. Through survival stories marked by constant endeavour and struggle, participants emerge as proactive agents attempting to navigate their precarious situation. Despite their efforts, however, they are unable to make positive changes of their working lives. Both women and men who became parents perceived parenthood as meaningful, offering a reprieve from their demanding circumstances, although single mothers in particular faced a heavy burden of caring responsibilities. In addition, power inequalities in the partner relationship could entail physical and or psychological abuse from their partners with severe mental ill-health consequences for the women. As other studies indicate [31, 32], alcoholism among the men also contributed to the violence and solitary living post-separation. In adulthood, the men could grieve for the end of their marriage, through which they lost contact with their children.

### II. A stable gendered working life in health-challenging jobs

Four participants, Jenny, Carola, Siri and Fredrik, maintained stable employment throughout their careers but experienced various health problems over time, due to their physically demanding and stressful working conditions. At 23, Jenny expressed remorse for leaving compulsory schooling and felt constrained as a young single mother in pursuing further education as financial obstacles forced her to withdraw.

The only thing I regret in these eight years is that I didn’t complete compulsory schooling, because now I won’t get the chance. It’s not possible if you don’t have a student allowance. I could take out a loan to study secondary education and higher education, but I’ll certainly not put myself in debt to complete eighth and ninth grade.

Reflecting at 24, Carola recounted a life shaped by caregiving duties since the onset of her first partnership and motherhood: “Yes, it’s as others say, first, it’s the mother who protects the boys, and then it’s the girl who takes care of everything”. She lamented her constrained educational opportunities as a young single mother. Like Sanna and Jenny, she embarked on adult education through the municipality to improve her compulsory schooling but found juggling studies with single parenthood too challenging. Alongside her role as a single mother, Carola, like Jenny, highlighted the inadequate financial support for single mothers pursuing education.

This “Knowledge boost” that everyone is talking about [a government labour market measure], you only get funding for a year. But I mean what can you study for *one year* to become something? Then you must take a student loan in the second year, and I’m not one to take loans.

The young women balanced career ambitions with gendered caregiving responsibilities, forced to prioritise financial security for the family. They became mothers early, navigating traditional gendered duties and partners who might be abusive. Carola emphasised the stability of single motherhood amid tumultuous family dynamics. Jenny and Carola obtained permanent positions in the care sector, but experienced monotonous, physically demanding and mentally stressful working conditions. Carola, like Jenny and other women in the study, constructed her working trajectory within a normative and altruistic feminine framework [26]. She described her limited agency to improve her work situation, alongside a higher purpose of taking care of her children. “I take the job I get and I’m content, that’s it; and I accept whatever I can get; I must, I can’t pick and choose. Not when you don’t have an education and in today’s situation, but I’m not sorry for that”. Despite enduring physical strain and injuries from her demanding job, Jenny in her 50s persisted with work. Carola, also in her 50s, was at the time of the interview on half-time sick leave due to an acute condition but aimed to keep working until retirement.

In her final interview, Siri, now 56, who had initially trained to work with disabled people, reflected on the meaningful but demanding nature of her three-decade career. Rising to the role of manager across multiple care facilities, she faced increasing workload and stress, culminating in her receiving disability pension due to exhaustion and depression. Siri lamented the escalating workload and stress in the care sector, citing high demand and minimal opportunities to influence her working conditions. She expressed frustration over the inability to provide optimal care due to lack of control.

It was too much at work and the demands… that we must do certain things in no time at all… and then the computer took over my work, so I couldn’t be with the users. Well, I could do *that*… and then it was like I did everything plus what we had to do on the computer. We had to make schedules and document who had been to the toilet and not, and who did not want to go to the store and why they didn’t want to go. Well, screw it, stop documenting! If someone doesn’t want to go to the store, so what? Do I have to write a paper [about that]? I mean, nobody writes what *I* do at home, and it’s *their* home. If they’re fine, let them be fine.

Throughout her life story, Siri exhibited agency and determination, which was often labelled as “stubbornness” by herself and others. Despite becoming a single mother at a young age, she pursued education to enter the care sector and left an abusive relationship. However, the demands of her work eventually overwhelmed her, and she reached a breaking point. “You know I’ve always dealt with everything, but one day it just stopped, it was impossible. It was like pulling down a roller blind. I went to the doctor and just cried”. Apart from attending brief job training sessions, Siri’s working life had come to an end, which she depicted as a turning point for improved health. Reflecting on her life journey, she renegotiated the meaning of her professional life.

So, you feel that it’s sad that you were so completely burned out that you couldn’t return to work, because it affects you financially. But, as I used to say, what is money in the big picture? If you aren’t well, nothing can replace that… So, my well-being has actually improved now.

The women who narrated the two first life paths frequently also discussed the challenges faced by their children and grandchildren, including struggles for special support in school. Poor mental health, including neuropsychiatric diagnoses and substance dependency, were also commonly reported.

Fredrik experienced a cycle of seasonal employment in his youth and beyond, often working double shifts but also facing periods of unemployment. These challenges led to persistent financial worries and an increase in stomach problems over time.

You wonder “do I have enough to pay the bills next month?” You can’t do anything else when you get home. If you lay down on the couch, you’ll fall asleep immediately. It’s just cooking [mostly sausages and macaroni] … and sleeping all weekend.

In a later interview, during his middle age, he expressed pride and enthusiasm about securing a permanent warehouse position, which he viewed as a turning point for improved well-being. While he remained in his childhood home and had not started his own family, he maintained strong ties with his siblings and a supportive circle of friends. He reported job satisfaction, overall contentment with life and good health.

Unlike the first life path, participants in this path secured stable employment, avoiding further unemployment-related hardships. Consequently, their stories lacked the instability and survival struggles of life path I, focusing instead on coping with demanding jobs and chronic diseases. Obtaining employment was seen as a significant achievement after enduring job insecurity, particularly for women who as single caregivers had to achieve financial stability. Nonetheless, in similarity with the first life path, many women faced stress-related health problems linked to the demands of work and in private life. Limited agency to improve their situation due to gendered responsibilities was often highlighted. Motherhood held meaning but being single earners meant longer working hours and less time spent with their children [23]. Receiving disability pension was portrayed as a pivotal turning point towards improved health.

### III. A self-realising gendered working life with improved health

The third working life path showcases stories of thriving careers and diverse accomplishments. Participants either secured permanent employment early on or pursued municipal adult education and additional education to advance their careers. These achievements were portrayed as turning points, fostering self-realisation and better health. David, Ola, Martin and Petra shared narratives marked by agency and resilience in reaching this stage. However, social support from parents and partners, and supportive senior colleagues, was also said to have played a significant role, empowering them to pursue their goals effectively.

David’s working trajectory took a significant turn when, after a prolonged period of unemployment in his youth, he landed a sales position in a large company despite lacking qualifications. This opportunity allowed him to advance to higher positions and pursue a successful business career. At 57, he enjoyed financial stability, in stark contrast to his impoverished upbringing. While recovering from a recent health issue, he maintained good health and an active lifestyle. Earlier struggles in a tumultuous relationship and being a separated father drained his energy, but now he had found happiness in marriage. Reflecting on leaving school, he revealed struggles with concentration after a family member’s death the year before, compounded by a lack of adult support.

They [his parents] were supportive, but [the close family member] was sick, and my mum couldn’t cope, and of course not dad either… They struggled to keep [the person] alive, so that was a daunting task for them. Somewhere I think that if the situation had not been this way… it would probably have all turned out different.

He pointed out that his teachers could have been more supportive, rather than dismissive, when he expressed his desire to leave school, and he compared his current life situation to his disadvantaged position in childhood.

Nowadays I’m able to provide my children with substantial financial stability. I don’t give them anything they shouldn’t have, but there was just no money when I was a child. Mum was at home caring for me and my siblings, and we had to live on what she had.

While David’s life had taken a positive turn, he believed completing secondary education would have significantly eased his journey. Reflecting, he emphasised the importance of schooling, stating it could have prevented many of his problems.

For the other participants, re-engaging in education played a significant role in improving their working life and health. Consistent with previous findings, their belief and agency were crucial in this process, as they expressed a strong sense of purpose and determination [33]. Ola dropped out of school in seventh grade. Although he said that the teachers thought “he was good at most things”, he stated that school was just not for him. At home, he described a difficult situation with a stepfather who terrorised the family and physically abused his mother. Following a fight, during which Ola hit his stepfather, he had to leave home at 16. He became temporarily homeless, moving between friends and struggling with unemployment and strained finances. Temporary homelessness in youth, especially after dropping out of school, increases vulnerability to risky behaviour and mental health problems [1]. He grappled with periods of unemployment, precarious jobs and health problems such as depressive episodes, headaches and sleeping problems. Ola realised he needed a change, declaring, “I won’t get anywhere if I stay here”. Transitioning to work as a chef marked a pivotal turning point. Supportive colleagues and opportunities to travel abroad boosted his confidence, inspiring him to pursue further education in the field. At 49, Ola reflected on his childhood family issues – alcohol abuse and domestic violence – and linked them to his struggles in school, citing a lack of adult support. He felt that periods of unemployment made him feel a burden to society. Now, Ola prioritises being a present and dedicated father. Like David, he viewed his life as a “series of uphill struggles”, demonstrating that his strong determination to achieve his goals had led to positive outcomes, representing a strong agentic narrative.

It is one’s own will, that “there is nothing that can stop me”. I set a goal and pursue it, and sooner or later I will reach it. I don’t stop until I get there, and then I find new goals. You must have both short- and long-term life goals and not just think day to day, because it was a lot like that when you were younger and there was no one there to explain.

During the Covid 19 pandemic, at 57, Ola’s business declined, and he became unemployed. He sought financial aid and housing while also battling a metabolic disease due to weight gain. However, in a follow-up a few years later, he had secured employment, lost weight and regained good health, overcoming the metabolic condition.

Martin recounted starting a new school in ninth grade where he felt misunderstood and discriminated against by his teachers, and therefore he lowered his ambitions. After completing compulsory schooling with low grades and experiencing brief periods of unemployment, he began working in his father’s company, where he received ample support and later secured a permanent position. Initially, he viewed his life path in line with the second life path: “It’s ok, but I’d rather be doing something else and sometimes I think ‘it’s crap to sit here and do the same thing over and over again, you’ll go crazy’. But at least you’ve got a job and I think you should be happy for that”. Before obtaining a permanent position, Martin struggled with excessive alcohol consumption, using it to cope with low self-esteem in social situations. He admitted he might have become heavily dependent on alcohol if not for his job. At 30, Martin developed a rheumatic condition from repetitive manual labour in cold conditions, prompting a life reassessment. Like Ola, Martin gained confidence in his work, receiving praise for his competence and reliability. This newfound confidence motivated him to pursue education, which became a turning point in his life – first qualifying for university, then training as a technician.

What was good about work was when you went to the construction site, and they said: “good it’s you”, well thank you very much! Then I felt appreciated and there was nothing that could mess that up; and it’s the same thing now. You want to be good at what you do so that people notice that this is carefully executed.

Martin also emphasised his strong determination to push his limits as a significant motivator when he re-entered education, ultimately attaining his current qualified position.

Yes, but you try to… push yourself to do things that you don’t always want to do. Especially with college, it made you wake up and realise that you can do things you never thought you could. It’s been something that has driven you along.

In his final interview, at 57, Martin reflected on the challenges of managing school due to his working-class background. Both his parents had negative school experiences, and his mother’s chronic disability made it hard for her to assist with his studies [22]. Like David and Ola, Martin portrayed himself as the master of his fate. Despite adversities, he achieved a meaningful working life and good health, aside from a rheumatic condition linked to his previous job.

Petra stands out as the only woman in the study who found self-realisation in her work without enduring long-term issues from poor working conditions. She completed further education, became an assistant nurse and pursued specialist courses. After securing a permanent position in the care sector in her youth, she initially followed the second life path. She valued the stability and balance that her job provided for her family life but faced temporary health issues such as high blood pressure and persistent headaches. Engaging with a trade union, due to her concerns about injustice and social inequality, she embarked on a parallel career. Eventually, her employer encouraged her to apply for an inspection job, which she successfully obtained.

In her final interview at 56, Petra expressed contentment with her work and social life. She cherished her long-term marriage, a healthy adult child and her role as a contact family for a young person in need through social services. Active in sports and enjoying good health, she credited further education as a turning point in her life, which made it possible to receive a more qualified position and motivated her to pursue advancement. Like the others, she attributed meaning to her confidence in overcoming obstacles and performing well, attributing this to the support of her parents: “I know my dad used to say, and mum also, that ‘you should think more of yourself than you do. You should always believe in yourself’, and that’s what I’ve done”. She remained committed to her work in the care sector, despite lower wages than in male-dominated jobs, driven by dedication to colleagues and a desire for supplementary income for travel. Reflecting on her decision to leave school early, she concluded:

If [my child] had said to me that “I am going to leave school” …You know, I would’ve done *everything* just to make him keep going. Yes, I would! Higher education paves the way for better jobs and financial stability.

In this path, participants demonstrated agency in improving their work situations and health. Through education and purposeful career decisions, and with social backing, they forged successful and health-promoting careers. Reflecting on their disadvantaged beginnings and past struggles, characterised by limited agency and poor health, they crafted their life stories within the cultural script of a redemption narrative [20, 34], highlighting their resilience in overcoming adversities. Nonetheless, gendered dynamics also influenced these trajectories. The men excelled in sales and technology sectors, while the lone woman worked in the care sector, which is lower paid [11] and has poorer working conditions [35].

## Discussion

This 40-year longitudinal study explored the life stories of early school leavers initially with NEET status, focusing on the evolution of their gendered working life trajectories linked to health, gender relations and agency. In line with McAdam’s theories, the life stories shifted from current situations and future aspirations in youth to reflections on the past in middle age [19, 20]. Hence, participants sought to create new meanings from their experiences, consolidate their identities and impart lessons to future generations, including their own children and other disadvantaged youth. The youth narratives revealed that participants, consistent with previous research, [36, 37] dropped out of school due to frustration and disengagement, influenced by low motivation, learning difficulties, experiences of failure, perceived lack of relevance, bullying and inadequate teacher support [36]. Family burdens, such as parental illness, loss of family members, alcohol misuse and family violence, also played a significant role in their leaving school [22, 37]. The neighbourhood seemed to be important, as the participants came from low socio-economic backgrounds, felt alienated from academic values and were part of peer communities where school dropout was not rare [38]. In the data, the young participants who dropped out of school in grades 8 or 9, and did not complete compulsory school, were most likely to end up in the most health impairing working life path.

Previous research has highlighted the negative health consequences of entering the labour market after early school drop-out [1–3]. Youth narratives in this study underscore the struggles of managing unemployment, securing jobs and enduring poor working conditions. The participants therefore experienced deteriorating mental health, including psychosomatic symptoms. Boyadjieva and Ilieva-Trichkova [39] have theorised how various factors constrain young people’s agency. Consistent with their ideas, the young participants in the study reported limited institutional support and felt unheard and unsupported by unemployment services, limiting their ability to improve their situation. Conversely, social support facilitated access to informal employment opportunities. However, some credited self-reliance and trusting their own ability to change their situation more than external resources.

Lister [8] describes personal agency as *strategic agency*, aimed at redirecting the life course by “getting out” of disadvantage and poverty, and *everyday agency*, focused on “getting by” with a burdensome life ( [8], p. 129–130). In line with her theory, the participants on the first two working life paths negotiated agency through survival narratives (about marginalisation, precarious and risky working conditions, gendered social problems, poverty, and ill-health) or coping narratives (about unfavourable working conditions and ill-health). Those on these more disadvantaged life paths relied on their agency or “stubbornness” to survive rather than change their situations. These paths were primarily narrated by women who had become sole caregivers and a man with a congenital chronic medical condition, whose vulnerability, according to Boyadjieva and Ilieva-Trichkova [39], restricted their agency. Structural problems, such as high unemployment rates and a lack of targeted interventions, further hindered access to education and employment [39]. During life, medical conditions developed which led to early labour market exit, particularly due to burnout among women in the care sector who faced high workloads and minimal decision-making opportunities. This supports Connell’s [10] theories about the impact of gendered processes on the labour market. Economic recessions led to gendered cuts in the workforce, and increased workloads in the welfare sector resulted in deteriorating mental health among employees in women-dominated jobs [40].

According to Lister’s theories [8], the most health-promoting working life path can be interpreted as forms of strategic agency, aimed at escaping disadvantage and poverty. Utilising Boyadjieva and Ilieva-Trichkova’s theories [39], this strategic agency manifests as self-improving actions intended to enhance employability in fulfilling roles, such as pursuing education to improve one’s competence.

As is demonstrated in other studies of vulnerable young people’s re-engagement in education [33], early employment opportunities were crucial, boosting self-confidence and motivating further education. Further education was interpreted as turning points in our study, and they were gendered as women seeking to return to education encountered barriers linked to lone motherhood and financial constraints. Shifts in social relations, including motherhood among young women, brought new meaning to life, but also led to exhausting juggling of children and work. Previous research emphasises the challenges single mothers encounter when returning to education [41, 42], while enabling programmes can create narratives of hope by helping them gain self-confidence to pursue their aspirations [42]. Despite their efforts, most participants, especially women and the chronically ill, faced lifelong poor labour market conditions due to early school leaving. Early retirement was described as a turning point for improved well-being.

Quantitative studies have repeatedly identified youth unemployment as well as other NEET positions as detrimental, especially to mental health [40]. In this qualitative study, we have helped to fill knowledge gaps by identifying the gendered health consequences of weak labour market attachment. While mental health problems are sometimes regarded as expressed differently by men and women [43], our study underscore the impact of gendered working life on the different health outcomes of working conditions between men and women. While the men were attracted to masculine coded jobs, with high risk of accidents, the women had to be the main caregiver in private life and worked in the welfare sector, which had the highest demands and the lowest level of control over the job situation. Such job situations are related to both depression and burnout among employees, who are mainly women [44, 45]. In line with the notion of “cycles of disadvantage” [46], adversity was also projected on to new generations, with participants reporting similar problems with their children and grandchildren.

### On the methods

This longitudinal narrative study provides unique insights into the complex situations of early school leavers during life. Through repeated interviews focused on participants’ working life trajectories and health, it highlights the importance of labour market conditions, gender relations, and agency in mitigating health issues. Conducted by the same researcher, the study likely fostered trust and facilitated discussion of sensitive topics. The participants – young people experiencing early unemployment – came from working class backgrounds in an industrial city in northern Sweden, suggesting potential relevance to similar groups in comparable contexts.

The first life path – “an unhealthy gendered working life in youth due to NEET status” – should be discussed from a contextual viewpoint. Although reviews have shown that NEET status or youth unemployment is related to mental ill-health in western countries [47, 48], other labour market regimes could provide different results. A Danish study [49] shows that four out of five young adults in NEET cannot be characterised as being at risk of social exclusion. The Danish flexicurity model has not been adopted in Sweden, due to criticism mainly from some trade unions of its increased flexibility.

Generalisation of our findings must take both time and place into account. For example, in relation to labour market policies, Denmark introduced a system of flexicurity that guarantees the availability of labour market measures, while Sweden has never accepted such insecurity in the labour market. In addition, the Swedish welfare model has been acknowledged worldwide for its universalism, comparatively generous replacement rates and extensive welfare services. Those born in the mid-1960s grew up and entered the labour market at a time when the Swedish welfare state was one of the most developed in the world. However, just before the age of 30, they encountered the deep recession of the 1990s, which was marked by sharply rising unemployment rates in combination with increased insecurity in the labour market, manifest as a shift from permanent to insecure temporary employment contracts.

The rise of neoliberalism, particularly in the 1990s, resulted in a restructuring of the Swedish welfare state, including the privatisation and marketisation of welfare services, along with stricter qualification criteria for benefits [50]. Consequently, young people in Sweden, as in many other western countries, are now growing up in a different welfare regime [50]. Thus, the situation in schools and for the unemployed is much more difficult today than it was when the NoSCo participants were young. For those growing up in Sweden of today, social inequalities in income and health have increased and our findings may not be directly comparable.

In addition, an earner-carer policy model aimed at the engagement of mothers and fathers in both paid and unpaid work is a hallmark of Nordic welfare states. Labour market participation is high among women in Sweden throughout working age. The welfare system provides preschools and free school lunches, and women are paid to do caring work. Even so, the proportion of women working part time is relatively high. Our findings must therefore be applied with caution to other welfare regimes.

### Policy implications

The study reveals that preventable factors such as family problems, lack of teacher support, bullying, low motivation and experiences of failure contributed to participants leaving school. This suggests that providing support to address diverse pupil needs, especially to those from disadvantaged families, and improved learning environments could help to counteract school dropout. To mitigate the adverse impacts of unemployment and challenging working conditions on young people’s health and well-being, labour market initiatives should prioritise job training and subsidised employment in healthy environments, while also offering training grants for re-entering education. It is crucial to create opportunities for young school leavers, including young mothers and those with chronic illnesses, to pursue self-realising educations and careers despite facing disadvantaged circumstances.

## Conclusions

Even in a welfare regime like Sweden’s in the early 1980s, early school leavers not in education, employment or training experienced class-related and gendered working and living conditions, which created unequal conditions for health. Despite Sweden’s active labour market policies and their own practices of agency, the participants still ended up NEET and with precarious working life paths. Labour market policies should prioritise reducing unemployment, combating precarious employment, creating job opportunities, providing training and subsidised employment in healthy environments, and offering grants to re-enter further education.

Our study highlights the need for further analyses of the contextual and gendered expressions of health among early school leavers throughout their lifetime, and of individual agency in various contexts for overcoming adversities.

## Data Availability

The data are not freely available. The Swedish Data Protection Act (1998:204) does not permit sensitive data on people (such as from our interviews) to be freely shared. Following ethical approval, the anonymous data set can be obtained on request from Umeå University following their secrecy examination.
